# A Survey on Explainable Artificial Intelligence (XAI) Techniques for Visualizing Deep Learning Models in Medical Imaging

**DOI:** 10.3390/jimaging10100239

**Published:** 2024-09-25

**Authors:** Deepshikha Bhati, Fnu Neha, Md Amiruzzaman

**Affiliations:** 1 Department of Computer Science, Kent State University, Kent, OH 44242, USA; neha@kent.edu; 2 Department of Computer Science, West Chester University, West Chester, PA 19383, USA; mamiruzzaman@wcupa.edu

**Keywords:** medical imaging, deep learning, machine learning, explainable AI, model interpretability

## Abstract

The combination of medical imaging and deep learning has significantly improved diagnostic and prognostic capabilities in the healthcare domain. Nevertheless, the inherent complexity of deep learning models poses challenges in understanding their decision-making processes. Interpretability and visualization techniques have emerged as crucial tools to unravel the black-box nature of these models, providing insights into their inner workings and enhancing trust in their predictions. This survey paper comprehensively examines various interpretation and visualization techniques applied to deep learning models in medical imaging. The paper reviews methodologies, discusses their applications, and evaluates their effectiveness in enhancing the interpretability, reliability, and clinical relevance of deep learning models in medical image analysis.

## 1. Introduction

Medical imaging (MI) is a cornerstone of modern healthcare, providing critical insights for diagnosing, treating, and monitoring various diseases. Traditionally, MI encompassed mesoscopic imaging techniques such as Magnetic Resonance Imaging (MRI), Computed Tomography (CT), and Positron Emission Tomography (PET). However, with recent technological advancements, the scope of MI has significantly broadened to include high-resolution histopathology, a vital subspecialty of pathology that focuses on examining tissue samples at microscopic and molecular levels. This area now leverages advanced techniques such as digital pathology and computational analysis.

The integration of artificial intelligence (AI) has revolutionized high-resolution histopathology, enhancing diagnostic accuracy and resolution. This expansion from traditional mesoscopic imaging to advanced microscopic techniques represents a significant evolution in MI. The field now includes histological, cellular, and molecular pathology, driven by AI advancements that support ongoing developments in diagnostic precision and therapeutic strategies. For instance, recent studies highlight the role of AI in analyzing electron microscopy images for disease monitoring [1,2] and improving deep learning applications in histopathology [3,4].

Traditional image analysis methods, reliant on handcrafted features and expert knowledge, are often time-consuming and prone to errors. Machine learning (ML) approaches, including Support Vector Machines (SVMs), decision trees, random forests, and logistic regression, have improved efficiency and accuracy in tasks such as image segmentation and disease classification. However, these methods still require manual feature selection and extraction. The advent of deep learning (DL) has transformed medical image analysis by automatically learning and extracting hierarchical features from large volumes of data [5,6,7,8,9,10,11,12,13]. This progress has provided healthcare professionals with valuable insights, enabling more accurate diagnoses and enhanced patient care.

Despite the impressive performance of DL models, challenges related to interpretability and transparency persist [14,15,16]. The opaque nature of these models raises concerns about their reliability in healthcare, where understanding diagnostic decisions is crucial. Interpretability in AI-driven healthcare models fosters trust and reliability by allowing practitioners to comprehend and verify model outputs. It ensures ethical and legal accountability, supports clinical decision-making, and helps identify biases and errors, enhancing fairness and accuracy.

Efforts to improve the interpretability of DL models in MI are ongoing, with researchers developing techniques to clarify model decision-making processes [17,18,19]. This paper contributes to this field through several key aspects:

1. Comprehensive Survey: We offer a thorough survey of innovative approaches for interpreting and visualizing DL models in MI, including a broad range of techniques aimed at enhancing model transparency and trust.
2. Methodological Review: We provide an in-depth review of current methodologies, focusing on post-hoc visualization techniques such as perturbation-based, gradient-based, decomposition-based, trainable attention (TA)-based methods, and vision transformers (ViT). We evaluate each method’s effectiveness and applicability in MI.
3. Clinical Relevance: We emphasize the importance of interpretability techniques in clinical settings, demonstrating how they lead to more reliable and actionable insights from DL models, thus supporting better decision-making in healthcare.
4. Future Directions: We outline future research directions in model interpretability and visualization, highlighting the need for more robust and scalable techniques that can handle the complexity of DL models while ensuring practical utility in medical applications.

**Figure 1:**
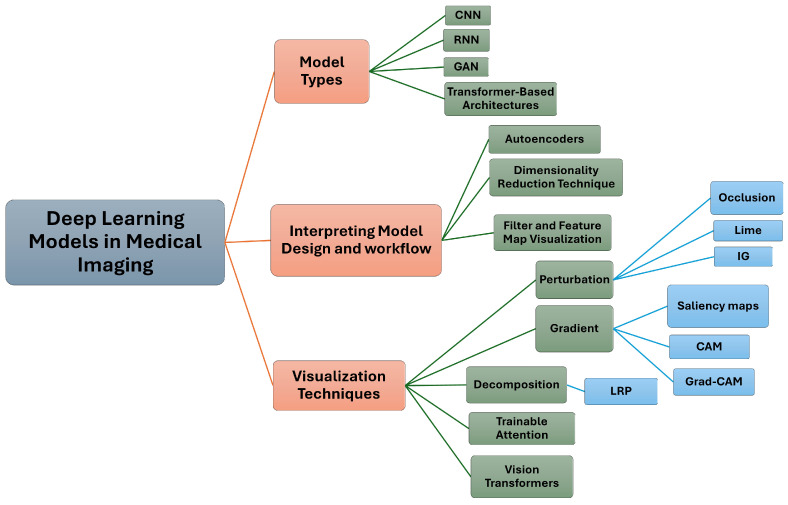
Overview of Deep Learning Models and Techniques in Medical Imaging: This diagram illustrates the main categories of deep learning models used in medical imaging, including model types, understanding model structure and functionality, and interpretation and visualization techniques. It highlights specific methods such as Convolutional Neural Networks (CNNs), Recurrent Neural Networks (RNNs), Generative Adversarial Networks (GANs), transformer-based architectures, autoencoders, Local Interpretable Model-agnostic Explanations (LIME), Integrated Gradient (IG), Gradient-Class Activation Mapping (Grad-CAM), and Layer-Wise Relevance Propagation (LRP), Attention and Vision Transformers.

Our survey covers innovative approaches for interpreting and visualizing DL models in MI. As illustrated in Figure 1, we explore various DL models and techniques, including Convolutional Neural Networks (CNNs), Recurrent Neural Networks (RNNs), Generative Adversarial Networks (GANs), transformer-based architectures, autoencoders, Local Interpretable Model-agnostic Explanations (LIME), Gradient-Class Activation Mapping (Grad-CAM), Layer-Wise Relevance Propagation (LRP), attention-based methods, and vision transformers (ViTs).

This review distinguishes itself by expanding the definition of medical imaging to include high-resolution histopathology and digital pathology, thus broadening the scope of traditional mesoscopic imaging techniques. This approach not only highlights recent advancements but also sets the stage for future research in interpretability and visualization within the expanding field of medical imaging.

The rest of this paper is divided into four sections, with multiple subsections within each of them. Section 3 is focused on interpreting model design and workflow. Section 4 Visualizing DL models in MI. Section 5 presents an overview of post-hoc interpretation and Visualization Techniques. A comparison of Different Interpretation Methods is discussed in Section 6 and concludes the work with current challenges and future directions in Section 8.

## 2. Research Methodology

A comprehensive review of explainable AI in medical image analysis was published by [20,21,22,23,24]. While this review covers a broad range of topics, some critical areas, such as research on trainable attention (TA)-based methods, vision transformers, and their applications, have been overlooked. Our review aims to fill this gap by providing an extensive overview of various domains within medical imaging, addressing key aspects such as Domain, Task, Modality, Performance, and Technique.

This research employs the Systematic Literature Review (SLR) method, which involves several stages. The research questions guiding this study are as follows:

- What innovative methods exist for interpreting and visualizing deep learning models in medical imaging?
- How effective are post-hoc visualization techniques (perturbation-based, gradient-based, decomposition-based, TA-based, and ViT) in improving model transparency?
- What is the clinical relevance of interpretability techniques for actionable insights from deep learning models in healthcare?
- What are the future research directions for model interpretability and visualization in medical applications?

The survey examines over 400 recent papers on explainable AI (XAI) in medical image analysis. Relevant contributions were identified using keywords like “deep learning”, “convolutional neural networks”, “medical imaging”, “surveys”, “interpretation”, “visualization” and “review”. Sources included ArXiv, bioRxiv and medRxiv, Google Scholar, Scopus, and Science Direct, focusing on titles. Studies without results on medical image data or using only standard neural networks with manually designed features were excluded. In cases of similar work, the most significant publications were selected.

The findings will be comprehensively presented, including a detailed description of the research methodology for replication. The literature search results, relevant articles, and their quality evaluations will be summarized in overview tables. Drawing from expertise in applying XAI techniques to medical image analysis, ongoing challenges, and future research directions will be discussed.

## 3. Interpreting Model Design and Workflow

Interpreting model design and workflow involves examining the hidden layers of convolutional neural networks (CNNs). This can be achieved through methods such as:

1. Autoencoders for Learning Latent Representations
2. Visualizing High-Dimensional Latent Data in a Two-Dimensional Space
3. Visualizing Filters and Activations in Feature Maps

### 3.1. Autoencoders for Learning Latent Representations

Autoencoders (AE) are DL models for unsupervised feature learning [25], with applications in anomaly detection [26], image compression [27], and representation learning [28]. They consist of an encoder creating latent representations and a decoder reconstructing images. Variants include variational autoencoders (VAE) and adversarial autoencoders (AAE). In medical imaging, AEs detect abnormalities by comparing input images with reconstructions and highlighting high reconstruction loss areas. For instance, VAE has reconstructed OCT retinal images to detect pathologies [29], and AAE has localized brain lesions in MRI images [30]. Convolutional AEs have detected nuclei in histopathology images by combining learned representations with thresholding [31].

### 3.2. Visualizing High-Dimensional Latent Data in a Two-Dimensional Space

CNNs produce high-dimensional features, making visualization challenging. Dimensionality reduction techniques like Principal Component Analysis (PCA) and t-Distributed Stochastic Neighbor Embedding (tSNE) simplify this data. PCA performs linear transformations, while tSNE [32] uses nonlinear methods to map high-dimensional data to lower dimensions. tSNE is effective for visualizing patterns and clusters, such as in abdominal ultrasound and histopathology image classification. The constraint-based embedding technique [33], using a divide-and-conquer algorithm to preserve k-nearest neighbors in 2D projections, has assessed deep belief networks separating brain MRI images of schizophrenic and healthy patients, though both tSNE and constraint-based embedding struggled with raw data separation.

### 3.3. Visualizing Filters and Activations in Feature Maps

A convolutional block extracts local features from input images through convolution filters, ReLU or GELU activations, and pooling layers. Filter visualization reveals CNN’s feature extraction capabilities, with initial layers capturing basic elements and later layers capturing intricate patterns. In medical imaging, filter visualization compares filters in Computer-Aided Detection (CAD) for 3D Computed Tomography (CT) images [9]. Larger filters offer more insights but require more memory. Feature map visualization, representing layer outputs after activation, highlights active features and can indicate training issues. It is used in tasks like skin lesion classification [34], fetal facial plan recognition in ultrasound [35], brain lesion segmentation in MRI [6], and Alzheimer’s diagnosis with PET/MRI [36].

## 4. Deep Learning Models in Medical Imaging

*Convolutional neural networks (CNNs)* are essential in DL for MI. CNNs are adept at processing X-rays, Computed Tomography (CT) scans, and Magnetic Resonance Imaging (MRI) through their hierarchical feature representations. Studies [5,6,7] have demonstrated their effectiveness in various medical image analysis tasks. For instance, in segmentation tasks, CNNs excel at delineating organ boundaries or identifying anomalies within medical images, providing valuable insights for accurate diagnosis and treatment planning. Recurrent Neural Networks (RNNs) excel in the temporal modeling of dynamic imaging sequences, such as functional MRI or video-based imaging, by capturing temporal patterns [37]. Generative Adversarial Networks (GANs) are valuable for image synthesis, data augmentation, anomaly detection, generating synthetic images and learning normal patterns [38,39,40].

### Transformer-Based Architectures

Transformer-based architectures, including bidirectional encoder representations from transformers (BERT) [41,42], and generative pre-trained transformer (GPT) [43], are emerging for tasks like disease prediction, image reconstruction, and capturing complex dependencies in medical images.

## 5. Interpretation and Visualization Techniques

In recent years, numerous explainable artificial intelligence (XAI) techniques have been developed to enhance the interpretability of DL models, particularly in MI. These techniques can be broadly categorized into Perturbation-Based, Gradient-Based, Decomposition, and Attention methods.

**Figure 2:**
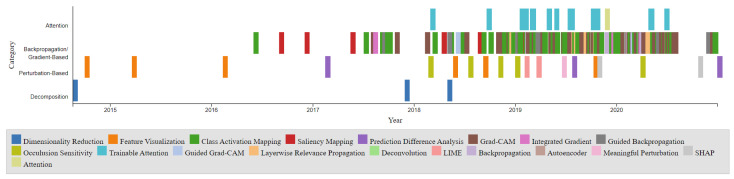
Timeline of XAI Technique Development in medical imaging applications.

The timeline presented in Figure 2 illustrates the development of these XAI techniques over the years. The points on the timeline are color-coordinated by respective categories, including Dimensionality Reduction, Feature Visualization, Class Activation Mapping, Saliency Mapping, Prediction Difference Analysis, Grad-CAM, Integrated Gradient, Guided Backpropagation, Occlusion Sensitivity, Trainable Attention, Guided Grad-CAM, Layerwise Relevance Propagation, Deconvolution, LIME, Backpropagation, Autoencoder, Meaningful Perturbation, SHAP, and Attention. Notably, the Gradient-Based category is the most densely populated, with CAM and Grad-CAM being among the most popular entries. The timeline also reveals a higher density of developments between 2017 and 2020. The comparison chart illustrates the visualization Techniques methods: Gradient-Based, Perturbation-Based, Decomposition-Based (LRP), and Trainable Attention Models in terms of model dependency, access to model parameters, and computational efficiency as shown in Table 1.

### 5.1. Perturbation-Based Methods

Perturbation-based methods evaluate how input changes affect model outputs to determine feature importance. By altering specific image regions, these methods identify areas that significantly influence predictions, typically visualized with heatmaps. As highlighted in recent surveys, perturbation-based XAI methods are crucial for exploring CNN models by systematically altering inputs and observing output changes, which is vital for understanding models used in safety-critical areas where transparency is essential [44]. Techniques like Integrated Gradients (IG), Local Interpretable Model-agnostic Explanations (LIME), and Occlusion Sensitivity (OS) are used in various domains, including breast cancer detection, eye disease classification, and brain MRI analysis (see Table 2). The table categorizes studies by domain, task, modality, performance, and technique. These methods also test model sensitivity to input variations, ensuring robust interpretations.

#### 5.1.1. Occlusion

Zeiler and Fergus [55] introduced an occlusion method to assess the impact on model output when parts of an image are obstructed. Kermany et al. [53] utilized this method for interpreting optical coherence tomography images to diagnose retinal pathologies. A major limitation of occlusion is its high computational demand, as it requires inference for each occluded image region, increasing with image resolution and desired heatmaps.

#### 5.1.2. Local Interpretable Model-Agnostic Explanations (LIME)

**Figure 3:**
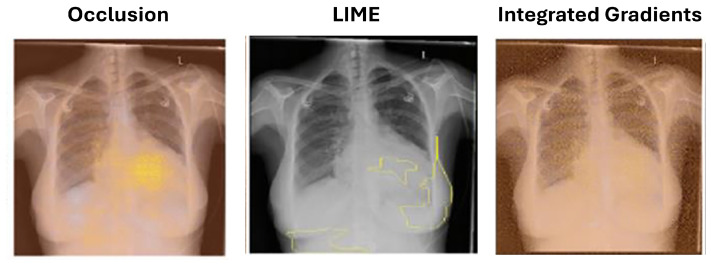
Example uses of perturbation-based attribution methods for model interpretability which shows the comparison of several approaches to interpretation for identifying congestive heart failure on chest X-rays (Seah et al., 2018) [54].

Recent studies emphasize the growing importance of local interpretation methods, such as LIME, which offer clear interpretability with lower computational complexity, making them suitable for real-time applications [56]. Ribeiro et al. [57] introduced local interpretable model-agnostic explanations (LIME) to identify superpixels (groups of connected pixels with similar intensities). Seah et al. [54] applied LIME to identify congestive heart failure in chest radiographs. LIME offers an advantage over occlusion by preserving the altered image portions’ context, as they are not completely blocked as shown in Figure 3.

#### 5.1.3. Integrated Gradients

Sundararajan et al. [47] introduced integrated gradients (IG) to measure pixel importance by computing gradients across images interpolated between the original and a baseline image with all non-values. Sayres et al. [46] found that model-predicted grades and heatmaps improved the accuracy of diabetic retinopathy grading by readers.

### 5.2. Gradient-Based Methods

**Figure 4:**
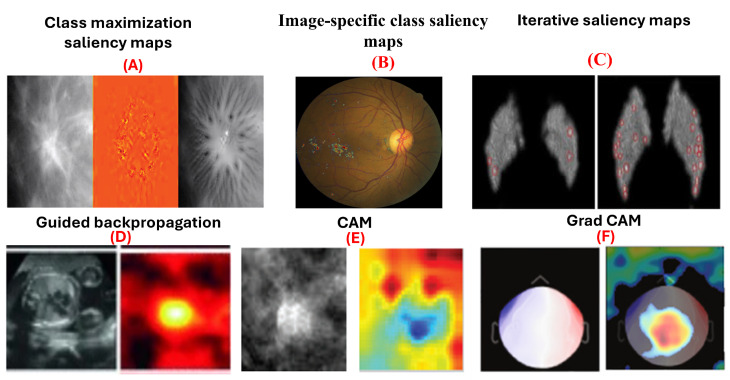
Examples of gradient-based attribution methods for model interpretability. (**A**) Class maximization visualization of malignant and benign breast masses on mammograms [13]. (**B**) Integrated gradients visualizing evidence of diabetic retinopathy on retinal fundus images [46]. (**C**) Visualization of malignant and benign breast masses [13]. (**D**) Guided backpropagation applied to ultrasound images for fetal heartbeat localization [8]. (**E**) Differentiation between benign and malignant breast masses in mammograms [10]. (**F**) Grad-CAM visualizations identifying discriminative regions in magnetoencephalography images for detecting eye-blink artifacts [59].

Backpropagation, used for weight adjustment in neural network training, is also employed in model interpretation methods to compute gradients. Unlike training, these methods do not alter weights but use gradients to highlight important image areas. The development of specialized deep learning networks like FISH-Net, which optimizes detection through innovative techniques such as rotated Gaussian heatmaps and noise refinement, highlights the potential of deep learning in achieving high precision and sensitivity in medical imaging tasks [58]. Figure 4 Shows examples of gradient-based attribution methods for model interpretability.

#### 5.2.1. Saliency Maps

Saliency maps, introduced by Simonyan et al. [60], use gradient information to explain how deep convolutional networks classify images. They are used in class maximization and image-specific class saliency maps. Class maximization generates an image-maximizing activation for a class, as in:

$$\arg\max S_{c}\left(I\right)-\lambda {∥I∥}_{2}^{2}$$

Yi et al. [13] applied this to visualize malignant and benign breast masses. Image-specific class saliency maps create heatmaps showing each pixel’s significance in classification, computed as:

$${\mathrm{Sal}}_{c}\left(x\right)=\frac{\partial F_{c}\left(x\right)}{\partial x}$$

Dubost et al. [61] used these maps in a weakly supervised method for segmenting brain MRI structures. Saliency maps have also been utilized in diagnosing heart diseases in chest X-rays [12], classifying breast masses in mammography [62] with accuracy ranging from 85% to 92.9%, and identifying pediatric elbow fractures in X-rays [63] with accuracy of 88.0% and area under curve (AUC) of 95.0%. Moreover, iterative saliency maps [64] enhance less obvious image regions by generating a saliency map, inpainting prominent areas, and iterating the process until the image classification changes or a limit is reached. This approach, applied to retinal fundus images for diabetic retinopathy grading, demonstrated higher sensitivity compared to traditional saliency maps. However, saliency has limitations. They do not distinguish if a pixel supports or contradicts a class, and their effectiveness diminishes in binary classification.

#### 5.2.2. Guided Backpropagation

Guided backpropagation, introduced by Springenberg et al. [65], builds on the saliency map approach by Simonyan et al. [60] and the deconvnet concept by Zeiler and Fergus [55]. It improves gradient backpropagation through ReLU layers, where negative activations are set to zero during the forward pass. Guided backpropagation discards gradients where either the forward activation or the backward gradient is negative, producing heatmaps that highlight pixels positively contributing to the classification.

In 2017, Gao and Noble [8] applied guided backpropagation to ultrasound images for fetal heartbeat localization. They found that the heatmaps remained consistent despite variations in the heart’s appearance, size, position, and contrast. Conversely, Böhle et al. [66] discovered that guided backpropagation was less effective for visualizing Alzheimer’s disease in brain MRIs compared to other methods. Similarly, Dubost et al. [67] achieved an intraclass correlation coefficient (ICC) of 93.0% in brain MRI detection using guided backpropagation. Wang et al. [68] obtained an average accuracy of 93.7% in brain MRI classification with this technique. Gessert et al. [69] reported an accuracy of 99.0% in cardiovascular classification using OCT images. Wickstrom et al. [70] achieved a 94.9% accuracy in gastrointestinal segmentation using endoscopy. Lastly, Jamaludin et al. [71] reported an accuracy of 82.5% in musculoskeletal spine classification using MRI images with guided backpropagation.

#### 5.2.3. Class Activation Maps (CAM)

Class Activation Mapping (CAM), introduced by Zhou et al. [72], visualizes regions of an image most influential in a neural network’s classification decision. CAM is computed as a weighted sum of feature maps from the final convolutional layer, using weights from the fully connected layer following global average pooling [73]. For a specific class *c* and image *x*:

$${\mathrm{CAM}}_{c}\left(x\right)=\sum_{k}w_{k}^{c}f_{k}\left(x\right)$$

This heatmap highlights regions most relevant for classification. CAM has been applied in various medical imaging applications, such as segmenting lung nodules in thoracic CT scans [74] and differentiating between benign and malignant breast masses in mammograms [10]. However, CAM’s effectiveness depends on the network architecture, requiring a global pooling (GAP) layer followed by a fully connected layer. While Zhou et al. [75] originally used GAP, Oquab et al. [76] demonstrated that global max pooling and log-sum-exponential pooling can also be used, with the latter yielding finer localization. Table 3 summarizes CAM’s effectiveness across different medical imaging tasks, covering domain tasks, modalities, and performance metrics.

#### 5.2.4. Grad-CAM

Grad-CAM, an extension of CAM by Selvaraju et al. [124], broadens its application to any network architecture and output, including image segmentation and captioning. It bypasses the global pooling layer and weights feature maps directly with gradients calculated via backpropagation from a target class. The gradients of the output for the class *c* concerning feature maps $A^{k}$ are averaged globally, multiplied by $A^{k}$, and passed through a ReLU activation to discard negative values:

$${\mathrm{Grad}-\mathrm{CAM}}_{c}\left(x\right)=\mathrm{ReLU}\left(\sum_{k}\left(\frac{1}{Z}\sum_{i}\sum_{j}\frac{\partial y^{c}}{\partial A_{ij}^{k}}\right)A^{k}\right)$$

Garg et al. employed grad-CAM visualizations to identify discriminative regions of magnetoencephalography images in the task of detecting eye-blink artifacts [59]. The authors found that the regions of the eye highlighted by grad-CAM are the same regions that human experts rely on. Table 4 summarizes the effectiveness of Grad-CAM across various medical imaging tasks, highlighting domains, tasks, modalities, and performance metrics.

### 5.3. Decomposition-Based Methods

Decomposition-based techniques for model interpretation focus on breaking down a model’s prediction into a heatmap showing each pixel’s contribution to the final decision. These techniques, such as LRP, have been widely applied across different domains.

#### Layer-Wise Relevance Propagation (LRP)

Layer-Wise Relevance Propagation (LRP), introduced by Bach et al. in 2015 [170], offers an alternative to gradient-based techniques like saliency mapping, guided backpropagation, and Grad-CAM. Instead of relying on gradients, LRP distributes the output of the final layer back through the network to calculate relevance scores for each neuron. This process is repeated recursively from the final layer to the input layer, generating a relevancy heatmap that can be overlaid on the input image. Further properties of LRP and details of its theoretical basis are given in (Montavon et al., 2017 [171]), and a comparison of LRP to other interpretation methods can be found in ([172,173,174]).

The relevance score $R_{i\leftarrow k}^{\left(l,l+1\right)}$ for a neuron *i* in layer *l* from a neuron *k* in layer l+1 is defined as:

$$R_{i\leftarrow k}^{\left(l,l+1\right)}=R_{k}^{\left(l+1\right)}\frac{a_{i}w_{ik}}{\sum_{h}R_{h\leftarrow k}^{\left(l,l+1\right)}}$$

The overall relevance score for neuron *i* in layer *l* is as follows:

$$R_{i}^{l}=\sum_{k}R_{i\leftarrow k}^{\left(l,l+1\right)}$$

**Figure 5:**
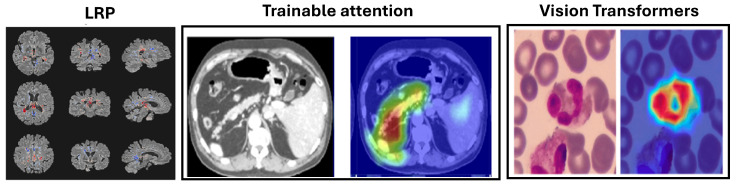
Example uses of decomposition-based attribution methods for model interpretability to show the Layerwise relevance propagation for model interpretation in diagnosing multiple sclerosis on brain MRI (Eitel et al., 2019) [175]. The Attention U-Net for organ segmentation in abdominal CT scans [177] and the areas upon which the model correctly focuses its predictions on the test images in explainable Vision Transformers model [178].

LRP has been applied in MI, such as diagnosing multiple sclerosis (MS) and Alzheimer’s disease (AD) using MRI scans. For MS, LRP heatmaps highlighted hyperintense lesions and affected brain areas [175] as shown in Figure 5, while for AD, they emphasized the hippocampal volume, a critical region for diagnosis [66]. LRP has been found to provide clearer distinctions compared to gradient-based methods and has been used in frameworks like DeepLight for linking brain regions with cognitive states [176].

### 5.4. Trainable Attention Models

Trainable Attention (TA) Mechanisms provide a dynamic approach to model interpretation by integrating attention modules into neural networks. Introduced for CNNs by Jetley et al. [179], these soft attention modules generate attention maps that highlight important image parts. They compute compatibility scores between local and global features $\langle l_{s},g\rangle$ using dot products and learned vectors *a*:

$$a_{s}=\frac{\exp(c_{s})}{\sum_{i=1}^{n}\exp\left(c_{s,i}\right)}$$

The output $g_{a}$ adjusts local features based on the attention map weights:

$$g_{a}=\sum a_{s}l_{s}$$

This method enhances signals from compatible features while reducing those from less compatible ones. Applications of attention mechanisms in medical imaging include the Attention U-Net for organ segmentation in abdominal CT scans [177], fetal ultrasound classification, and breast mass segmentation in mammograms [180]. Additionally, they have improved melanoma lesion classification [181] and osteoarthritis grading in knee X-rays [182]. In cancer diagnostics, the CACNET method integrates attention mechanisms with Mask R-CNN to enhance nuclear segmentation and reduce noise interference, significantly improving the accuracy of CAC identification [183]. Attention-weighted RL (AWRL) models combine self-attention mechanisms with value function approximation to effectively filter out irrelevant features and enhance decision-making processes in complex tasks [184]. The Trainable Feature Matching Attention Network (TFMAN), incorporating non-local and channel attention, exemplifies how trainable attention mechanisms can augment representation capabilities in CNNs for image super-resolution [185]. Though optimal configurations are application-specific, attention mechanisms are valued for their interpretability and performance enhancement.

Table 5 provides an overview of various studies using TA models, highlighting the domains, tasks, modalities including MRI and histology, and performance metrics such as accuracy and F1-score, demonstrating the broad applicability and effectiveness of TA models in MI.

### 5.5. Vision Transformers

Vision Transformers (ViTs) have emerged as a prominent alternative to convolutional neural networks (CNNs) in medical imaging. Unlike CNNs, which use local receptive fields to capture spatial hierarchies, ViTs employ self-attention to model long-range dependencies and global context [42,196,197]. By partitioning images into fixed-size patches treated as a sequence, ViTs utilize transformer encoder layers, effectively capturing complex anatomical structures and pathological patterns.

ViTs have been employed to automate the Tanner-Whitehouse 3 (TW3) algorithm for bone age assessment, achieving clinically interpretable results with predictive accuracy comparable to that of experienced orthopedic surgeons [198]. ViTs have also demonstrated improved performance in diagnosing conditions such as tuberculosis, pneumothorax, and COVID-19 by leveraging self-supervision and self-training through knowledge distillation [199]. In the domain of medical image registration, ViTs have been shown to enhance the accuracy of volumetric image alignment significantly, outperforming traditional methods by capturing long-range spatial dependencies [200]. ViTs have also been applied to 3D cryogenic electron tomography (cryoET) data, with CryoViT outperforming CNNs in segmenting complex organelles like mitochondria, particularly when training data are limited [201].

ViTs have shown superior performance in segmentation, classification, and detection tasks, achieving high accuracy in segmenting tumors and organs in MRI and CT scans, as reflected in Dice scores. Their interpretability is enhanced through attention maps, gradient-based methods, and occlusion sensitivity, which aid in visualizing model predictions. These advancements highlight ViTs’ potential to improve diagnostic accuracy and provide deeper insights into medical image analysis, as discussed in Table 6. The areas upon which the model correctly focuses its predictions on the test image are presented in Figure 5. The regions of focus identified by the ViT model exhibit significant overlap with the areas of white blood cells [178].

## 6. Comparison of Different Interpretation Methods

### 6.1. Categorization by Visualization Technique

Visualization techniques in DL can be categorized based on their application and effectiveness. Table 7 summarizes various visualization techniques used in DL for interpretability. It categorizes methods based on their tasks, body parts, modalities, accuracy, and evaluation metrics. This table highlights that techniques like CAM and Grad-CAM are effective for image classification and localization across different modalities such as X-ray and MRI, achieving high accuracy. LRP is noted for its accuracy in segmentation tasks, while IG is utilized for classification with notable AUC-Receiver Operating Characteristic (ROC) scores. Attention-based methods improve performance and interpretability by focusing on relevant regions, whereas perturbation-based methods assess model robustness. LIME provides model-agnostic explanations, and trainable attention models dynamically enhance feature focus.

### 6.2. Categorization by Body Parts, Modality, and Accuracy

Table 7 provides a concise overview of imaging techniques categorized by anatomical context (Body Parts). It lists various modalities such as MRI, X-ray, and ultrasound, and highlights specific techniques used for different body parts. For instance, CAM and Grad-CAM are prominent in brain imaging with high accuracy, while LRP and attention-based methods excel in breast imaging. The table also emphasizes the adaptation of methods to address challenges such as speckle noise in ultrasound imaging.

The advanced segmentation techniques discussed include the improved V-net algorithm, which enhances liver and tumor segmentation using distance metric-based loss functions, and the LViT model, which integrates medical text annotations to improve segmentation performance in multimodal datasets [238,239]. Additionally, Transformers have been applied broadly in medical image analysis, significantly improving performance in tasks such as segmentation and classification [240].

**Figure 6:**
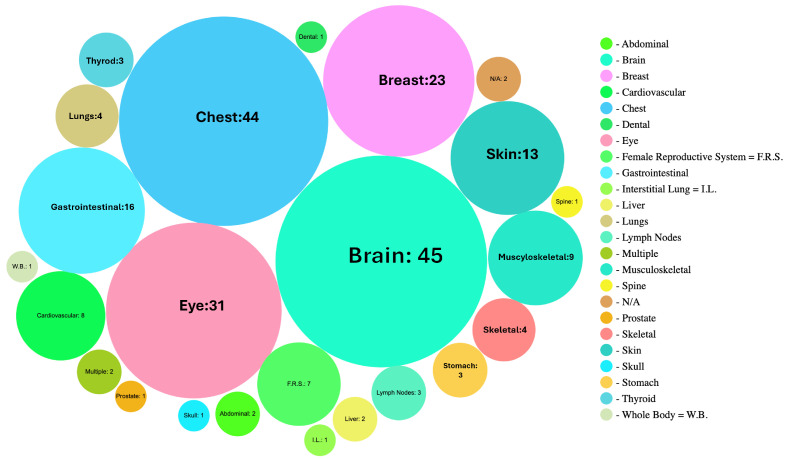
Bubble chart provides a concise overview of imaging techniques categorized by anatomical contexts.

The imaging techniques covered in the studies include CT, dermatoscopy, diabetic retinopathy (DR), endoscopy, fundus photography, histology, histopathology, mammography, magnetoencephalography (MEG), MRI, OCT, PET, photography, ultrasound, and X-ray. These studies spanned from 2014 to 2020, with the majority published in 2019 and 2020, as shown in Figure 6.

### 6.3. Categorization by Task

This section organizes the techniques and their applications across different tasks, highlighting performance metrics and examples for clarity. Table 8 organizes interpretability techniques according to their tasks, including classification, segmentation, and detection. It details the applications, performance metrics, and specific examples for each task. Techniques like CAM, Grad-CAM, and TA models are effective for classification tasks, providing high accuracy and AUC-ROC scores. LRP and Integrated Gradient are highlighted for segmentation tasks, with metrics like dice similarity coefficient (DSC) and intersection over union (IoU). Detection tasks benefit from methods such as saliency maps and CAM, with metrics including mean average precision (mAP) and sensitivity.

## 7. Current Challenges and Future Directions

### 7.1. Current Challenges

Despite significant advancements, several challenges remain in the interpretability and visualization of DL models in MI:

Scalability and Efficiency: Many interpretability methods, such as occlusion and perturbation-based techniques, are computationally intensive. This limits their scalability, especially with high-resolution medical images that require real-time analysis.

Clinical Integration: Translating interpretability techniques into clinical practice requires seamless integration with existing workflows and systems. This includes ensuring that the visualizations are intuitive for non-technical healthcare practitioners and that they provide actionable insights.

Robustness and Generalization: Interpretability methods must be robust across diverse patient populations and medical imaging modalities. Models trained on specific datasets might not generalize well to other contexts, leading to potential biases and inaccuracies in interpretations.

Standardization and Validation: There is a lack of standardized metrics and benchmarks for evaluating the effectiveness of interpretability methods. Rigorous validation in clinical settings is essential to establish the reliability and trustworthiness of these techniques.

Ethical and Legal Considerations: The opacity of deep learning models raises ethical and legal concerns, especially in healthcare where decisions can have critical consequences. Ensuring transparency, accountability, and fairness in AI-driven diagnostics is paramount.

### 7.2. Future Directions

To address the challenges and enhance the field of medical image interpretability, future research should focus on the following directions:

Expansion to High-Resolution Histopathology: As the field of medical imaging evolves to include high-resolution histopathology and digital pathology, future research should explicitly consider these advanced techniques. This includes developing interpretability methods tailored to microscopic and molecular-level images, which require different approaches compared to traditional mesoscopic imaging methods.

Development of Lightweight Methods: Creating computationally efficient interpretability techniques that can handle high-resolution images and deliver results in real-time is crucial. This involves optimizing existing methods and exploring new algorithmic approaches that balance accuracy with computational efficiency.

Enhanced Clinical Collaboration: Collaborative efforts between AI researchers, clinicians, and medical practitioners are necessary to design interpretability methods that are both clinically relevant and user-friendly. This could include the development of interactive visualization tools that allow clinicians to intuitively explore and understand model outputs.

Robustness to Variability: Developing interpretability techniques that are robust to variations in imaging modalities, patient demographics, and clinical conditions is essential. This requires extensive training on diverse datasets and continuous validation across different settings to ensure that methods remain effective and reliable in varied contexts.

Establishment of Standards: Creating standardized benchmarks and validation protocols for interpretability methods will aid in objectively assessing their effectiveness and reliability. This includes the development of common datasets and metrics for comparative evaluations to facilitate consistency and transparency in the field.

Ethical Frameworks: Integrating ethical considerations into the design and deployment of interpretability methods is critical. This involves ensuring that models are transparent, explainable, and free from biases, as well as addressing privacy and data security concerns. Ethical frameworks will support the responsible use of AI in medical imaging.

Hybrid Approaches: Combining different interpretability techniques, such as perturbation-based and gradient-based methods, can provide more comprehensive insights into model behavior. Hybrid approaches can leverage the strengths of various methods, enhancing overall interpretability and providing a more nuanced understanding of model decisions.

By addressing these directions, the field can advance towards more effective, reliable, and clinically relevant interpretability methods in medical imaging, paving the way for better integration of AI technologies in healthcare.

## 8. Conclusions

In conclusion, the integration of interpretability and visualization techniques into DL models for MI holds immense potential for advancing healthcare diagnostics and treatment planning. While significant progress has been made, challenges related to scalability, clinical integration, robustness, standardization, and ethical considerations persist. Addressing these challenges requires ongoing collaboration between AI researchers, clinicians, and healthcare practitioners. Future research should focus on developing efficient and clinically relevant interpretability methods, establishing standardized evaluation protocols, and ensuring ethical and transparent AI applications in healthcare. By overcoming these hurdles, we can enhance the trustworthiness, reliability, and clinical impact of DL models in MI, ultimately leading to better patient outcomes and more informed clinical decision-making.

## Figures and Tables

**Table 1:** Comparison chart that illustrates the visualization techniques methods.

Attributes	Perturbation	Gradient	Decomposition	Trainable Attention Models
Model Dependency	Model-agnostic	Differentiable	Model-specific	Model-specific
Access to Model Parameters	No	Yes	Yes	Yes
Computational Efficiency	Slower	Faster	Varies	Varies

**Table 2:** Overview of Various Studies Using Perturbation-Based Methods in Medical Imaging.

Domain	Task	Modality	Performance	Technique	Citation
Breast	Classification	MRI	N/A	IG	[45]
Eye	Classification	DR	Accuracy: 95.5%	IG	[46]
Multiple	Classification	DR	N/A	IG	[47]
Chest	Detection	X-ray	Accuracy: 94.9%, AUC: 97.4%	LIME	[48]
Gastrointestinal	Classification	Endoscopy	Accuracy: 97.9%	LIME	[49]
Brain	Segmentation, Detection	MRI	ICC: 93.0%	OS	[50]
Brain	Classification	MRI	Accuracy: 85.0%	OS	[51]
Breast	Detection, Classification	Histology	Accuracy: 55.0%	OS	[52]
Eye, Chest	Classification, Detection	OCT, X-ray	Eye Accuracy: 94.7%, Chest Accuracy: 92.8%	OS	[53]
Chest	Classification	X-ray	AUC: 82.0%	OS, IG, LIME	[54]

**Table 3:** Performance metrics of various Medical Imaging tasks across different modalities using CAM.

Domain-Task	Modality	Performance	Citation
Bladder Classification	Histology	Mean Accuracy: 69.9%	[77]
Brain Classification	MRI	Accuracy: 86.7%	[78,79]
Brain Detection	MRI, PET, CT	Accuracy: 90.2–95.3%, F1: 91.6–94.3%	[80,81]
Breast Classification	X-ray, Ultrasound, MRI	Accuracy: 83.0–89.0%	[82,83,84,85]
Breast Detection	X-ray, Ultrasound	Mean AUC: 81.0%, AUC: Mt-Net 98.0%, Sn-Net 92.8%, Accuracy: 92.5%	[86,87,88,89]
Chest Classification	X-ray, CT	Accuracy: 97.8%, Average AUC: 75.5–96.0%	[90,91,92,93,94,95,96,97]
Chest Segmentation	X-ray	Accuracy: 95.8%	[98]
Eye Classification	Fundus Photography, OCT, CT	F1: 95.0%, Precision: 93.0%, AUC: 88.0–99.0%	[99,100,101,102,103]
Eye Detection	Fundus Photography	Accuracy: 73.2–99.1%, AUC: 99.0%	[104,105,106,107,108]
Gastrointestinal (GI) Classification	Endoscopy	Mean Accuracy: 93.2%	[109,110,111,112]
Liver Classification, Segmentation	Histology	Mean Accuracy: 87.5%	[113,114]
Musculoskeletal Classification	MRI, X-ray	Accuracy: 86.0%, AUC: 85.3%	[115,116]
Skin Classification, Segmentation	Dermatoscopy	Accuracy: 83.6%, F1: 82.7%	[117,118]
Skull Classification	X-ray	AUC: 88.0–93.0%	[119]
Thyroid Classification	Ultrasound	Accuracy: 87.3%, AUC: 90.1%	[120]
Lymph Node Classification, Detection	Histology	Accuracy: 91.9%, AUC: 97.0%	[121]
Various Classification	CT, MRI, Ultrasound, X-ray, Fundoscopy	F1: 98.0%, Accuracy: 98.0%	[122,123]

**Table 4:** Performance metrics of various Medical Imaging tasks across different modalities Using Grad-CAM.

Domain-Task	Modality	Performance	Citation
Brain Classification	MRI	81.6–94.2% accuracy	[125,126,127,128,129,130]
Brain Detection	Ultrasound	94.2% accuracy	[131]
Breast Classification	MRI	91.0% AUC	[132]
Breast Segmentation	Histology	95.6% accuracy	[133]
Cardiovascular	CT, X-ray, Ultrasound	81.2–92.7% accuracy, AUC (81.0–96.3%)	[134,135,136,137]
Chest Classification	X-ray, CT, Histology	72.0–99.9% accuracy, AUC (70.0–97.9%)	[138,139,140,141,142,143,144,145,146,147,148,149]
Dental Classification	X-ray	85.4% accuracy, 92.5% AUC	[150]
Eye Classification	Fundus, OCT	81–97.5% accuracy, AUC (48.1–99.2%)	[151,152,153,154,155]
Gastrointestinal (GI) Classification	CT, Endoscopy, Histology, MRI	86.9–93.7% accuracy	[156,157,158,159,160]
Musculoskeletal	X-ray	74.8–96.3% accuracy	[161,162,163,164]
Thyroid Classification	CT	82.8% accuracy, 88.4% AUC	[165]
Whole-Body Scans	MRI	R2 value of 83.0%	[166]
Liver segmentation	CT scans	96% accuracy LiTS	[167]
Brain Tumor Detection	MRI images	98.52% accuracy	[168]
Breast Cancer	DISH and FISH images	97% accuracy	[169]

**Table 5:** Overview of Studies Using Trainable Attention Models in Medical Imaging.

Domain	Task	Modality	Performance	Citation
Brain	Detection	MRI	Accuracy: 76.5%	[186]
Brain	Detection, Classification	MRI	CC: 61.3–64.8%, RMSE: 1.503–5.701	[187]
Breast	Classification	X-ray	Accuracy: 85.0%, AUC: 89.0%	[188]
Breast	Segmentation	Mammo	Accuracy: 78.4%, F1: 82.2%	[189]
Breast	Classification	Histology	Accuracy: 90.3, AUC: 98.4%	[190]
Chest	Detection	X-ray	Accuracy: 73.0–84.0%	[191]
Chest	Classification	CT	Accuracy: 87.6%	[192]
Chest	Segmentation	MRI	Accuracy: 91.3%	[193]
Eye	Detection	Fundus Photography	Accuracy: 96.2%, AUC: 98.3%	[180]
Gastrointestinal (GI)	Classification	Histology	Accuracy: 88.4%	[194]
Skin	Dermatoscopy			[195]
Skin	Classification	Dermatoscopy	Average Precision: 67.2%, AUC: 88.3%	[181]
Female Reproductive System, Stomach	Classification, Segmentation	CT, Fetal Ultrasounds	Ultrasound Classification: Accuracy: 97.7–98.0%, F1: 92.2–93.3%, CT Segmentation: Recall: 75.1–83.5%	[177]
Skeletal (Joint)	Classification	X-ray	Accuracy: 64.3%	[182]

**Table 6:** Overview of Studies Using Vision Transformers in Medical Imaging.

Domain	Task	Modality	Performance	Citation
Stomach	segmentation	CT, MRI	Dice Score: 77.5%, Hausdorff distance: 31.7%	[202]
Brain, Pancreas, Hippocampus	segmentation	MRI, CT	Dice Scores: Brain: 87.9%, Pancreas: 83.6%, Hippocampus: 88.1%	[203]
Bile-duct	segmentation	Hyperspectral	Average Dice Score: 75.2%	[204]
Brain	segmentation	MRI	Dice Scores: Enhancing Tumor Region: 78.7%, Whole Tumor Region: 90.1%, Regions of Tumor Core: 81.7%	[205]
Brain, Spleen	segmentation	MRI, CT	Dice Score: 89.1%	[206]
Eye, Rectal, Brain	segmentation	Fundus, Colonoscopy, MRI	Average Dice Score: 91.7%	[207]
Eye	segmentation	Pathology	Dice Score: 78.6%, F1: 82.1%	[208]
Multi-organ	segmentation	Colonoscopy, Histology	Average Dice Score: 86.8%	[209]
Aorta, Gallbladder, Kidney, Liver, Pancreas, Spleen, Stomach	segmentation	MRI, CT	Average Dice Score: 78.1–80.4%	[210,211,212,213,214,215]
Heart	segmentation	MRI	Average Dice Score: 88.3%	[216]
Skin, Chest	segmentation	X-ray, CT	Average Dice Score: Skin: 90.7%, Chest: 86.6%	[217]
Rectal	segmentation	Colonoscopy, Histology	Average Dice Score: 91.7%	[218]
Kidney	segmentation	CT	Dice Score: 92.3%	[219]
Heart	segmentation	Echocardio- graphy	Dice Score: 91.4%	[220]
Brain	segmentation	MRI	Dice Score: 91.3–93.5%	[221,222]
Teeth	segmentation	X-ray	Dice Score: 92.5%	[223]
Breast	classification	Ultrasound	Accuracy: 86.7%, AUC 95.0%	[224]
Lung	classification	Microscopy	Accuracy: 97.5%	[225]
Eye	classification	Fundus	Accuracy: 95.9%, AUC: 96.3%	[226,227]
Chest	classification	Ultrasound	Accuracy: 93.9%	[228]
Chest	classification	X-ray	Average AUC: 93.1%, Accuracy: COVID: 98.0%, Pneumonia: 92.0%	[229,230,231,232]
Lung	classification	CT	F1: 76.0%	[233]
Chest	classification	X-ray, CT	Overall Accuracy: 87.2–98.1%, F1: 93.5%	[234,235,236,237]

**Table 7:** Comparison of Visualization Techniques.

Visualization Technique	Task	Body Parts	Modality	Accuracy	Evaluation Metric
CAM	Image classification and localization	Brain, chest, abdomen	X-ray, MRI, CT scans	85.0–95.0%	Accuracy for classification; IoU for localization tasks
Grad-CAM	Image classification and localization	Brain, chest, abdomen	X-ray, MRI, CT scans	85.0–95.0%	Accuracy for classification; IoU for localization tasks
LRP	Segmentation, classification	Brain, liver, lungs	MRI, CT scans	90.0%	Dice coefficient for segmentation accuracy
IG	Image classification	Breast, lung, spine	X-ray, MRI	80.0–92.0%	AUC-ROC for classification
Attention-based	Image classification, object detection	Brain, chest	X-ray, MRI	5.0% to 10.0%	Accuracy for classification; mAP for object detection
LIME	Local explanations for model predictions	N/A	N/A	N/A	Task-specific metrics
Gradient-based	Visualize feature importance	N/A	N/A	N/A	Feature importance metrics, SHAP values, Grad-CAM++
Vision Transformer	Dynamically attend to relevant features	Various body parts	Various modalities	N/A	Task-specific metrics

**Table 8:** Techniques Organized by Task.

Task	Techniques	Application	Performance Metrics	Examples
Classification	CAM, Grad-CAM, Attention, ViTs	Disease diagnosis, organ identification	Accuracy, AUC-ROC, Precision, Recall	Disease Diagnosis: High AUC for cancer detection (e.g., mammograms); Organ Identification: CAM for liver segmentation or brain MRI; ViTs: High accuracy in lung and breast cancer classification
Segmentation	LRP, IG, ViTs	Tumor segmentation, anatomical structure delineation	Dice Similarity Coefficient (DSC), Intersection over Union (IoU)	Tumor Segmentation: Accurate tumor boundary delineation; Anatomical Structure: IG for cardiac structure in CT scans; ViTs: High DSC scores in brain and stomach segmentation
Detection	Saliency maps, CAM, Attention, ViTs	Lesion detection, nodule localization	Mean Average Precision (mAP), Sensitivity, Specificity	Lesion Detection: Saliency maps for skin cancer detection; Nodule Localization: CAM for lung nodule detection in CT scans; ViTs: Improved lesion detection in various modalities

## Data Availability

All data were presented in main text.
